# Characterization of Tibetan Medicine *Zuota* by Multiple Techniques

**DOI:** 10.1155/2013/198545

**Published:** 2013-09-05

**Authors:** Xiao-Yan Zhao, Mei Sun, Jing-Xia Wang, Yun-Zhang Xu, Yuan Liu, Zhi-Feng Zhang, Lu-Yang Lu

**Affiliations:** Institute of Ethnic Medicine, Southwest University for Nationalities, Chengdu 610041, China

## Abstract

*Zuota* is regarded as the king of Tibetan medicine. However, due to the confidentiality of this precious medicine, the scientific characterization of *Zuota* is very scarce, which limits the pharmacology and biosafety studies of *Zuota*. Herein, we collected four different *Zuota* samples from Tibet, Qinghai, Gansu, and Sichuan and characterized them by multiple techniques. Our results showed that *Zuota* was mainly an inorganic mixture of HgS, sulfur, and graphite. Morphologically, *Zuota* samples were composed of nanoparticles, which further aggregated into microsized particles. Chemically, the majorities of *Zuota* were S and Hg (in the forms of HgS and pure sulfur). All samples contained pure sulfur with orthorhombic crystalline. *Zuota* from Qinghai province had different HgS crystalline, namely, hexagonal crystalline. The others were all face-centered cubic crystalline. Carbon in *Zuota* NPs was in the form of graphite. The implication to future studies of *Zuota* was discussed.

## 1. Introduction

Tibetan medicine has a history of over 3800 years [1]. Known as the king of Tibetan medicine, *Zuota* is the transliteration of Tibetan language “*Renqing Ouqu Zuozhu Qinmu*.” Here, “*Zuo*” means refining, and “*ta*” means grey powder. Thus, *Zuota *means burning to powder. *Zuota* has been used as an essential and key component of *Renqing* series drugs in Tibetan medicine for 1200 years [2, 3]. *Zuota* cannot be used as medicine alone, but it shows magic effect when used as supplementary material to other medicines [4]. Based on *Zuota*, many drugs have been developed, such as *Qi Shi Wei Zhen Zhu Wan*, *Er Shi Wu Wei Zhen Zhu Wan*,* Er Shi Wu Wei Shan Hu Wan*,* Er Shi Wu Wei Song Shi Wan*,* Er Shi Wu Wei Er Cha Wan*, *Renqing Chang Jue*,* Renqing Mang Jue*,* Zuo Zhu Da Xi*, and* Zhi Tuo Jie Bai Wan. *These medicines are applied in treating digestive diseases, cardiovascular and cerebrovascular diseases, hepatic and gall diseases, food poisoning, pa-disease, leprosy, and so on [5–10]. *Zuota* shows very good performance in treating diseases of skin, knuckle, marrow, and sclerotin [2, 3]. *Zuota* is also beneficial in replenishing blood, activating blood, and prolonging the life according to Tibetan medicine theory [2, 3, 11, 12]. The proposed effects of *Zuota* in these medicines are enhancing the pharmaceutical effects and reducing the toxicity [2, 3]. Recently, *Zuota* has been collected in the Chinese Intangible Cultural Heritage [13].

However, nearly nothing is known about *Zuota* from the modern scientific perspective, because* Zuota* is always treated as the top secret of Tibetan medicine. According to the disclosure, *Zuota* is prepared from Hg by Tibetan special processing method, which takes 300 steps and 40 days [14]. During the processing, many precious animal medicines, 8 heavy metal elements, and 8 mineral substances are added. Despite the mystery of *Zuota*, the major starting material (Hg) is a toxic element undoubtedly according to modern medicine [15, 16]. Therefore, the biosafety of *Zuota* has always been concerned [11, 12, 17, 18]. Besides, the pharmacology of Hg in *Zuota* is still a mystery [19–21]. To address the pharmacology and toxicity of *Zuota*, the first and essential step is to reveal the physicochemical properties of *Zuota*. 

Unfortunately, such information is still scarce to date [22–26]. There are only several pilot studies available in literature. Zeng et al. determined the Hg contents in *Zuota* by high performance liquid chromatography (HPLC) [22]. Lan et al. analyzed the chemical components in *Zuota*, where the elements were quantified and the morphology was recorded [23]. Similar results were also reported by Yan et al. [24, 25]. In addition, the graphite carbon in *Zuota* was analyzed by Yan and Ma showing the existence of graphite [26]. However, current studies did not concern the chemical states of elements in *Zuota*. And the comparison among *Zuota* samples from different sources has not been performed.

Herein, we collected four different *Zuota* samples and characterized them by various techniques. The morphology of *Zuota* samples was characterized by scanning electron microscopy (SEM). The element contents were analyzed by X-ray fluorescence (XRF). The inorganic components were further analyzed by X-ray photoelectron spectroscopy (XPS), Raman spectroscopy, X-ray diffraction (XRD), and Thermogravimetric analysis (TGA). The infrared (IR) spectroscopy was adopted to provide the preliminary information on the chemical groups of *Zuota* samples. Our results showed that *Zuota* was mainly an inorganic mixture of HgS, sulfur, and graphite, forming nanoparticles (NPs). The implication to future studies of *Zuota* as nanomedicine was discussed.

## 2. Materials and Methods

### 2.1. Materials

 The *Zuota* samples were kindly provided by four institutes in China. The sources were listed in Table 1. The main protocols in preparing these samples were well documented in the literature [14]. The samples were sealed in plastic tubes and stored in desiccator before characterization.

### 2.2. Measurements

The morphology of *Zuota* was characterized by SEM (Quanta 200FEG, FEI, the Netherlands) at different magnifications. The elemental compositions of *Zuota* samples were characterized by XRF (S4-Explorer, Bruker, Germany). The element compositions of *Zuota *samples were analyzed by XPS (Axis Ultra, Kratos, UK) using an Al anode. During collecting the whole spectra, the scanning step was set as 1000 meV and the power was 150 W. For analyzing each element, the scanning step was set as 100 meV and the power was 150 W. Raman spectra of *Zuota* samples were recorded on a Renishaw micro-Raman instrument equipped with a Melles-Griot 35 mW He : Ne laser (laser excitation at 633 nm, 1.75 mW power, ×50 objective, laser spot size ~2 *μ*m × 2 *μ*m, and 50 s collection time). The crystallization information of *Zuota* samples was obtained by XRD (D8 Advance, Bruker, Germany). The scanning was performed under 26°C from the 2*θ* of 5° to 80° with a step size of 0.02° and a step time of 17.7 s. The weight loss of *Zuota* samples in air was analyzed by TGA (SDT 2900, Thermal, USA). Around 5 mg *Zuota* sample was analyzed at a ramp of 10°C/min. The balance gas was nitrogen (40 mL/min) and the sample gas was air (60 mL/min). The samples were extracted by ethanol for 24 h and the supernatant was analyzed on HPLC (Agilent technologies 1200, USA). The chemical groups of *Zuota *samples were analyzed by IR spectra (Magna-IR 750, Nicolet, USA).

## 3. Results and Discussion

### 3.1. Morphology

**Figure 1:**
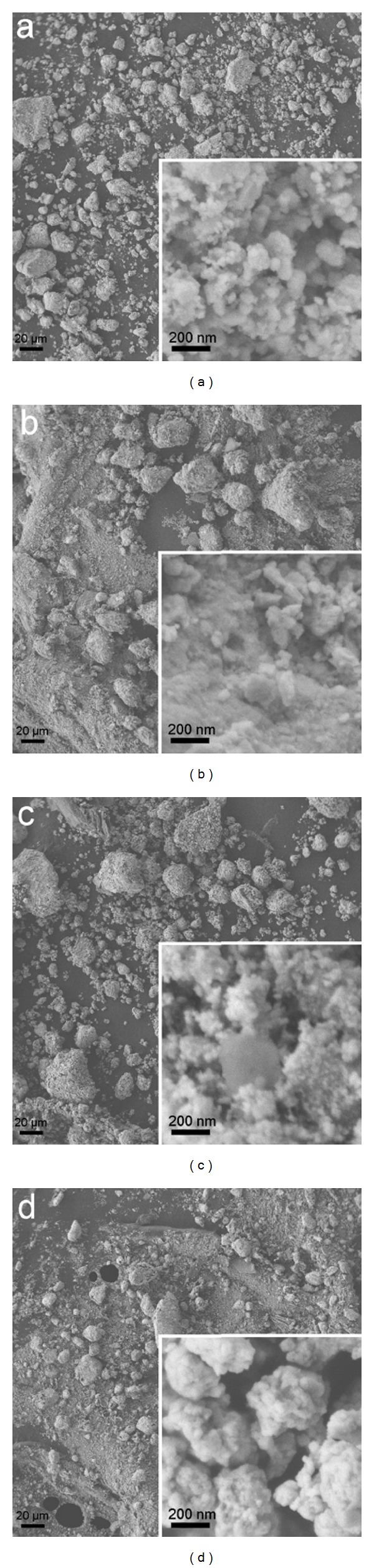
SEM images of *Zuota* samples (×300). (a) S1; (b) S2; (c) S3; (d) S4. The sects were images of higher magnification (×50,000).

*Zuota* samples displayed different morphologies under SEM (Figure 1). Generally, *Zuota* samples were majorly composed of very small NPs with other morphologies [24, 25]. The diameters of these NPs varied among the four samples; in the range of 10–50 nm. *Zuota* NPs further assembled into larger particles. The aggregation of NPs was the characteristics of *Zuota* samples, thus, we did not disperse the samples before analyses. Interestingly, in S2 and S4, there was another morphology observed, seeming like sludge. *Zuota* NPs were decorated on top of the sludge. This might indicate that the crystallization of S2 and S4 was not completed yet.

 The finding of NPs in *Zuota* samples was kind of surprise. We did not suggest that Tibetan had developed this “nanomedicine” in purpose. However, this nanomorphology might provide a possible route to explain the magic effects of *Zuota*, since NPs have been proven to have different pharmaceutical effect comparing to the bulk ones [27–29]. The nanomorphology might also provide a simple route to prepare analogues of *Zuota*. Scientists have developed various techniques to prepare NPs. The modern techniques hold great potential in facilitating the preparation of *Zuota*-like NPs, which will definitely promoting the production and applications of *Zuota*. Thus, the chemical components of *Zuota* need to be distinguished and quantified.

### 3.2. Element Concentrations

The compositions from XRF were expressed as the percentages of elements. As shown in Table 2, the most abundant component was S and Hg. There were about 50 wt% of S and 50% of Hg in the samples. S2 had the lowest S content, namely, 42.4%. S2 and S4 had higher Hg contents (55.3 wt% for S2 and 50.3 wt% for S4). Other elements, including Mg, Al, Si, K, Ca, Fe, Cu, Zn, Rb, Sn, and Pb, were also detected with much lower contents. It is worth to note that XRF provides the result of deep scanning, which stands for the average information of sample. When converting the weight ratio to atom ratio, *Zuota* samples showed very high S : Hg ratios (6.8, 4.8, 7.1, and 5.8 for S1–4, resp.). For the compounds of Hg and S, the common forms were HgS and Hg_2_S. Thus, there was excess S existing in *Zuota* samples, which might be in the form of pure sulfur. Actually, when performing the Brunauer-Emmett-Teller (BET) analyses, the pure sulfur evaporated during the pretreatment. Light yellow powders formed during the evaporation, which indicated the existence of pure sulfur. The abundant Hg and S detected in *Zuota* samples were consistent with the chemical composition of starting materials, mercury, and sulfur.

### 3.3. Inorganic Components

**Figure 2:**
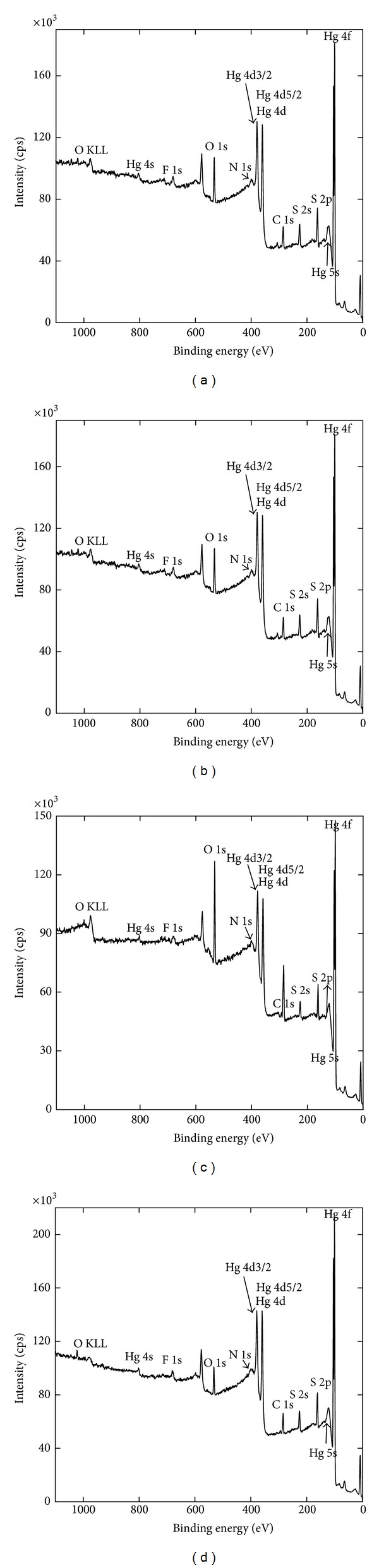
Full XPS spectra of *Zuota *samples. (a) S1; (b) S2; (c) S3; (d) S4.

According to XRF, *Zuota* samples were mostly inorganic. We further utilized several techniques to characterize the inorganic components of *Zuota* samples. Unlike XRF, XPS only detects the surface (several nanometers in depth) and provides the surface compositions (except H). As shown in Figure 2 and Table 3, several light elements were detected (C, N, and F). Other elements, such as O, Hg, Se, and S, were identified in Figure 2. Obviously, the element species in the surface were different from those in the whole sample. Again, *Zuota* samples showed high S : Hg ratios (2.6, 2.4, 2.2, and 2.5 for S1–4, resp.). These values were much lower than those from XRF. Nevertheless, S was still excess, implying the existence of pure sulfur.

**Figure 3:**
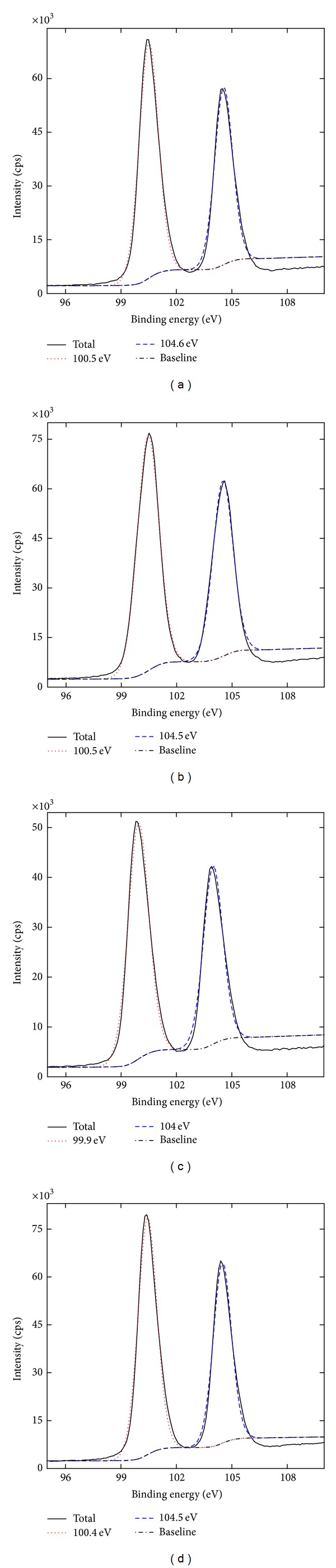
Hg4f spectra of *Zuota *samples. (a) S1; (b) S2; (c) S3; (d) S4.

XPS is powerful in characterizing the chemical state of element. As shown in Figure 3, two typical peaks were observed in the Hg4f spectra. The peak around 100 eV indicated the Hg4f_5/2_. The peak around 104.5 eV was the Hg4f_7/2_ XPS peak. The intensities of the four samples have nearly identical patterns. According to the literature, the enhanced doublet indicated that Hg was oxidized [30]. Therefore, we inferred that Hg was in the form of HgS, which was consistent with the XRD results.

**Figure 4:**
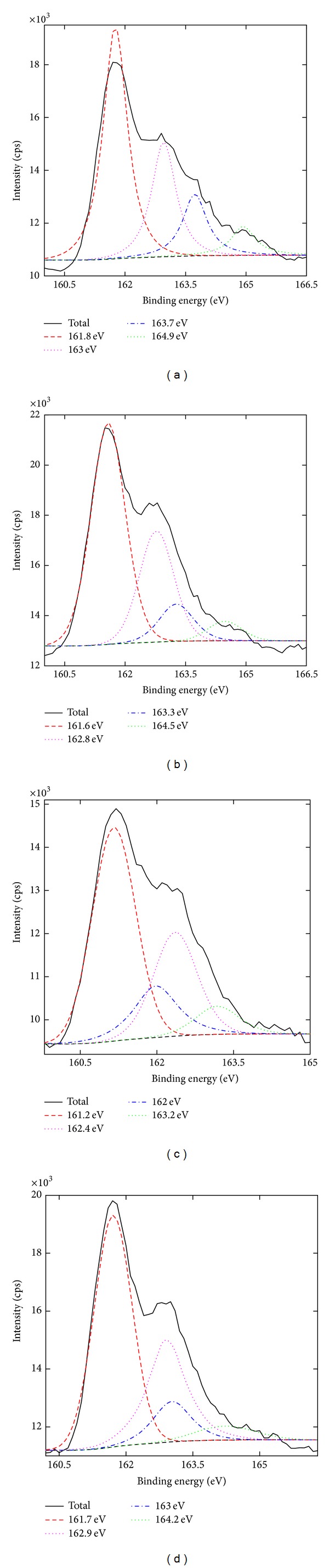
S2p spectra of *Zuota* samples. (a) S1; (b) S2; (c) S3; (d) S4.

Two groups of S2p peaks were observed in the S2p spectra (Figure 4). In each group, the area of the bigger one was set as twice of that of the smaller one. The binding energy of the bigger one was set as 1.2 eV lower than that of the smaller one [31]. The peaks around 161.5 and 162.7 eV indicated the S^2−^, for example, HgS. The peaks around 163.0 and 164.2 eV stood for pure sulfur. The components of the four samples showed similar patterns. S2 had the lowest pure sulfur, while S1 had the highest. The content of S^2−^ was higher than that of Hg, which implied that there might be other sulfides in *Zuota *samples.

**Figure 5:**
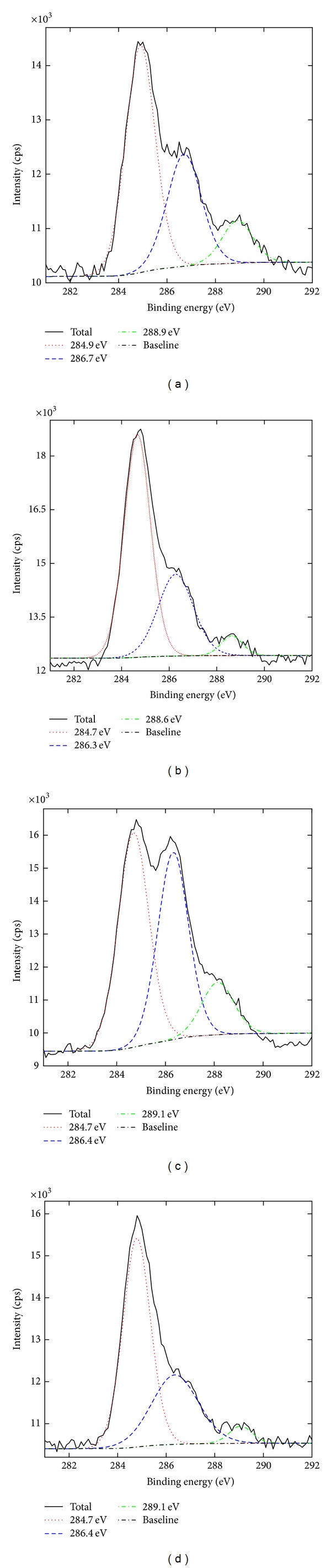
C1s spectra of* Zuota* samples. (a) S1; (b) S2; (c) S3; (d) S4.

Another element detected in XPS was C, which might be introduced by heating and carbonizing of the organic starting materials. Clearly, some C atoms were oxidized (Figure 5). S3 had the highest oxidative degree. 40.96% of C was oxidized to form C–O bonds. 12.01% of C atoms in S3 was further oxidized to form C=O bonds. S2 and S4 were less oxidized. More than 60% of C in S2 and S4 remained at 0 valent. Apparently, the C1s XPS spectra of *Zuota* samples were very similar to those of sp2 carbon materials, such as graphene oxide and carbon NPs [32, 33]. Hence, we adopted Raman spectroscopy to characterize the graphite structure in* Zuota* samples.

**Figure 6:**
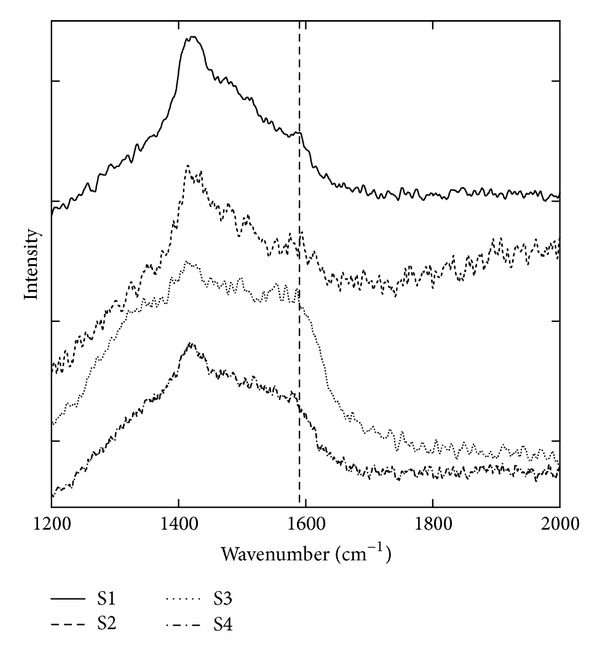
Raman spectra of *Zuota* samples. The position of 1590 cm^−1^ was indicated by the dash line.

Typically, the signal around 1590 cm^−1^ (G-band) indicates the graphite structure of carbon materials. As shown in Figure 6, there were meaningful signals around 1590 cm^−1^ in all *Zuota* samples. S3 had the highest C content and showed more distinguishable G-band in Raman spectrum. The G-band signals were recognized in S1, S2, and S4, but the relative intensities were lower. Another typical signal of graphite is D-band. D-band was observed in all *Zuota* samples. It is well known that D : G ratio is an indicator for the integrity of graphite structure. S1 had the highest D : G ratio, indicating that the graphite structure was the most disordered one. There might be more defects in S1. S3 showed the lowest D : G ratio, suggesting that graphite structure was more integrated in S3.

**Figure 7:**
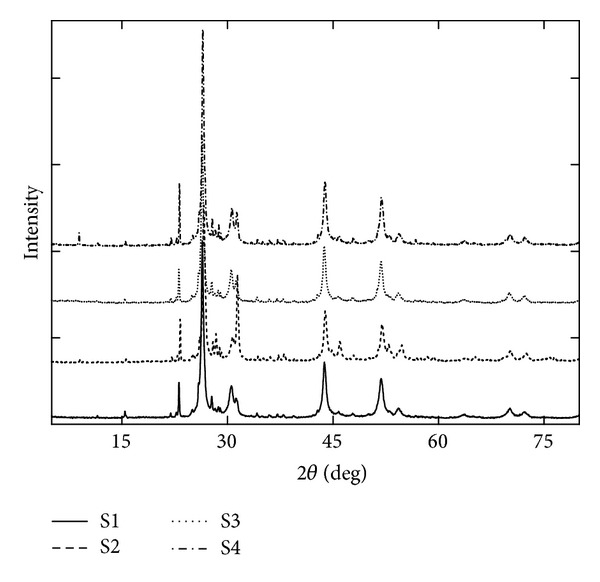
XRD spectra of *Zuota* samples.

The abundant inorganic components in *Zuota* samples were evidenced in the XRF and XPS analyses. Such components might be in the form of crystals. Therefore, we collected the crystallization information of *Zuota* samples by XRD (Figure 7). Despite the detailed information, there were two distinct characteristics reflected by the XRD analyses. Firstly, XRD confirmed the existence of pure sulfur in *Zuota* samples. All samples contained pure sulfur with orthorhombic crystalline. The sulfur crystals were all face-centered. The cell parameters were as follows: *a* = 10.43700, *b* = 12.84500, *c* = 24.36899, and *α* = *β* = *γ* = 90°. Secondly, S2 had different HgS crystalline, namely, hexagonal crystalline. HgS in S2 was primitive hexagonal crystal. The cell parameters were as follows: *a* = *b* = 4.13170, *c* = 9.44510, *α* = *β* = 90°, and *γ* = 120°. The others (S1, S3, and S4) were all face-centered cubic crystalline. The cell parameters were as follows: *a* = *b* = *c* = 5.85370 and *α* = *β* = *γ* = 90°. The results here suggested that although all samples were the so-called *Zuota*, they did have significant difference in physiochemical properties. The different crystalline structures might come from the various processing protocols, because each institute has its own tricks in processing *Zuota* beyond sharing the same major steps. The influence of such difference on the bioactivity of *Zuota* samples is worthwhile to investigate in the future.

**Figure 8:**
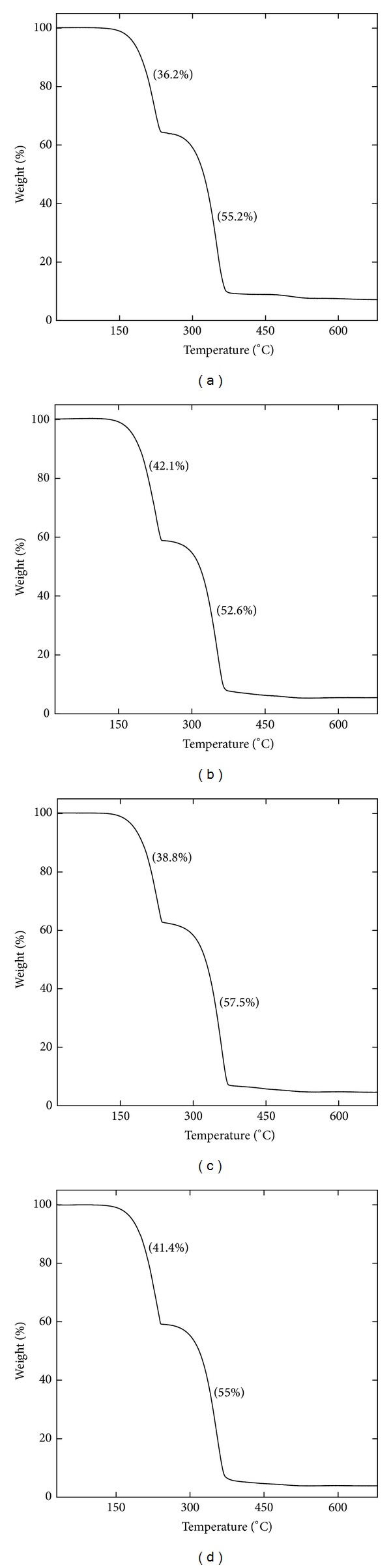
TGA curves of *Zuota* samples. (a) S1; (b) S2; (c) S3; (d) S4.

The existence of HgS and pure sulfur was complementarily reflected by TGA. As shown in Figure 8, all samples had two stages of weight loss. The first stage occurred at about 225°C and the second stage occurred at 350°C. It is very hard to make an accurate conclusion on what were burned during the TGA analyses. Probably, S and C were burned at the first stage, where the temperature was quite close to the burning point of S (about 232°C). Hg was evaporated at the second stage (close to the boiling point of Hg). The ratio of the two stages was very close to the weight ratio of S : Hg in XRF analyses (Table 2). Some other metals were left in the ash (less than 10%). 

### 3.4. IR Spectra

**Figure 9:**
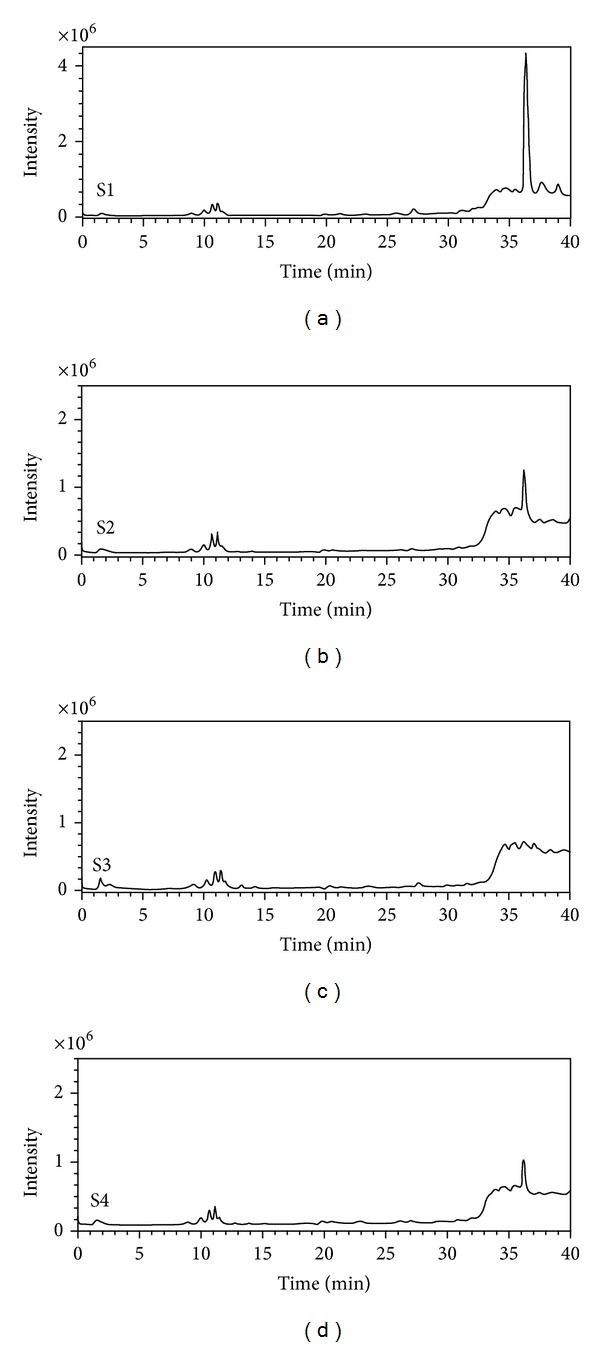
HPLC analyses of organic matters in *Zuota* samples.

**Figure 10:**
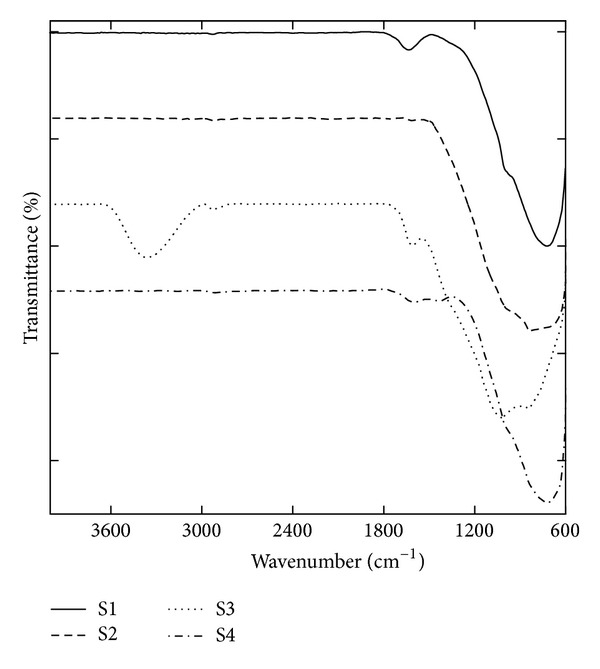
IR spectra of *Zuota* samples.

Although the majority of *Zuota *samples were inorganic substances, we did observe some organic substances in the HPLC (Figure 9). The existence of organic substances in* Zuota* was reasonable, because many animal and plant drugs are added during the production of *Zuota* [14, 23]. However, the system was too complicated to analyze the organic substances in detail [23]. As a preliminary characterization, we used IR spectroscopy to explore the potential organic components (Figure 10). There were very tiny peaks observed at 2923 cm^−1^. This indicated that there were C–H bonds in *Zuota *samples. Interestingly, S3 had a broad band at 3363 cm^−1^, which suggested the existence of –OH or –COOH. This was consistent with the C1s XPS spectrum, that S3 showed the highest oxidation degree. It seems to us that S3 had more H atoms, since the stronger band at 3363 cm^−1^ and peak at 2923 cm^−1^ were presented. The peaks around 1620 cm^−1^ were assigned to C=O bonds. The signals around 1000 cm^−1^ might indicate the C–O bonds. The peaks at about 717 cm^−1^ (S1, S2, and S4) and at 840 cm^−1^ (S2 and S3) might be the C–H bonds on aromatic rings. Indeed, such characterization was very preliminary. For future studies, detailed characterizations of the organic components of *Zuota* samples are highly encouraged.

## 4. Conclusion

Overall, we characterized four *Zuota* samples from different sources. *Zuota* samples were found to be inorganic NPs. The existence of graphite, sulfur, and HgS was confirmed by multiple techniques. Referring to IR spectra, there are also some functional groups containing oxygen atoms and hydrogen atoms in *Zuota *samples. We believe that our results will benefit the understanding of *Zuota* from modern scientific perspective. The nanoeffect of *Zuota* NPs and the analogues of *Zuota* NPs by nanotechnology are highly encouraged to investigate in future.

## Figures and Tables

**Table 1:** Sources of* Zuota* samples.

Sample	Source
S1	Tibet Traditional Medicine Factory, Lhasa, China
S2	Qinghai Tibetan Medical Hospital, Xining, China
S3	Gansu Institute of Tibetan Medicine, Gannan, China
S4	Aba Tibetan Medical Hospital, Maerkang, China

**Table 2:** Element concentrations in *Zuota* samples. The data were obtained from XRF and expressed as percentage of weight.

	S1 (wt%)	S2 (wt%)	S3 (wt%)	S4 (wt%)
MgO	0.373	0.228	—	0.194
Al_2_O_3_	0.196	0.176	0.099	0.318
SiO_2_	0.461	0.446	0.243	0.589
SO_3_	50.196	42.442	52.378	46.780
K_2_O	0.174	0.337	0.256	0.295
CaO	0.132	0.113	—	0.270
Fe_2_O_3_	1.050	0.516	0.418	0.379
CuO	0.499	0.196	0.361	0.321
ZnO	0.267	0.133	0.165	0.253
Rb_2_O	0.080	0.102	0.079	0.087
SnO_2_	0.161	—	—	—
Hg	45.985	55.312	45.762	50.311
PbO	0.427	—	0.241	0.203

**Table 3:** Element contents in *Zuota* samples. The data were obtained from XPS and expressed as percentage of weight.

	S1 (wt%)	S2 (wt%)	S3 (wt%)	S4 (wt%)
O	7.72	5.01	13.27	4.88
C	8.66	8.02	17.34	7.21
N	2.10	2.52	3.43	2.07
F	1.16	1.42	1.17	—
Hg	44.57	48.21	38.82	48.89
Se	17.08	16.63	12.40	17.63
S	18.71	18.20	13.57	19.32
